# Evaluation of nutrition education in school curriculum of Nepal: a descriptive content analysis

**DOI:** 10.1186/s40795-026-01453-7

**Published:** 2026-08-07

**Authors:** Aashma Dahal, Neeti Bhat, Tirtha Man Shrestha, Bibechana Sapkota

**Affiliations:** 1 Department of Public Health and Community Medicine, Madan Bhandari Academy of Health Sciences, Hetauda, Nepal; 2 Department of Physiology, Madan Bhandari Academy of Health Sciences, Hetauda, Nepal; 3 Department of General Practice and Emergency Medicine, Maharajgunj Medical Campus, Kathmandu, Nepal

**Keywords:** Curriculum development, Dietary choices, School education, Content analysis, Curricular comparison, Nutrition education

## Abstract

**Background:**

School going children and adolescents are at critical period of cognitive, physical and emotional development. Poor dietary behavior developed during these years can hinder healthy adulthood. Integration of nutrition education into curriculum could prove as a cost-effective intervention for behavior change in a low resource country like Nepal.

**Aim:**

This curriculum analysis study aims to assess the inclusion of nutrition related education in school curricula of Nepal in grades of 6–8, its duration and depth, identify gaps, and propose strategies for effective integration of such topics into the existing curricula and help the children to make informed dietary choices.

**Methods:**

The researchers used PRECEDE part of PRECEDE_PROCEED and Taba’s model of curriculum development for the review. First a narrative review was undertaken to understand nutritional landscape of Nepalese children from national Demographic Health Surveys and Global Nutrition Reports. Then, the contents of Basic Education Curriculum of Nepal (revised in 2020) for Grades 6–8 and all official textbooks published by the Curriculum Development Centre of Nepal were analyzed. The evaluation of curricula content was based on research questions given by the evaluation models used. Then for comparison of national curricula with global standards, it was compared with international curricula: (1) Alberta Health Services (Canada) and (2) Community Voices for Health (American University, USA) and UK government curriculum.

**Results:**

The curriculum of grade 6–8 was found to be nearly aligned from the national needs and priorities only in health education subject. In other subjects like social studies and sciences the topics on nutrition were completely missing. Pedagogical and assessment techniques were traditional. Overall, there is more scope for an enhanced curricum aligned with global standards of teaching and learning.

**Conclusion:**

Curricular revision is required prioritizing national nutritional needs among children and should inform healthy choices through creative pedagogical approaches.

## Introduction

Educating children and adolescents about healthy behavior at an early age is a proven method for influencing their lifestyle choices as adults. Translating nutrition knowledge into terms that are needed, understood, culturally acceptable, socially acceptable, and adopted is a major challenge for the upliftment of nutritional status. Regrettably, preparing individuals to lead a healthy life through informed nutritional choices has not received the same importance as other technical fields such as science and mathematics. This practice keeps the concept of primordial level of prevention in health neglected [1].

By leveraging intricate interdependence of health and education, we could bring more impact on health promotion through mutual reinforcement [2]. One such successful innovative program in Nepal is “school mid-day meals", where students are given nutritious lunch in schools, which aims to improve their education and health outcomes [3]. Despite this, it is imperative to acknowledge that these interventions have not fully achieved their goals as there has been a narrow focus on mitigating immediate hunger through basic sustenance whilst neglecting the educational component since childhood [4]. Research suggests that at least 50 hours of nutrition education per year is required to support behavior change [5]. Also, nutrition education in schools can lead to informed nutritional behaviors, has shown to reduce childhood obesity and improved academic performance [6]. The approach from “nutrition into education” could benefit from a complementary and parallel approach of “education into nutrition” for more sustainable and impactful changes. While nutrition into education emphasizes inclusion of nutrition related programs in schools like iron folic acid distribution to adolescents in schools and mid-day meals. education into nutrition implies educational interventions for health promotion. The latter are more cost effective and yet more effective means to improve the status of nutritional indicators in the long run [4]. This study identifies shortcomings in the existing secondary level curriculum of Nepal and provides strategies for curricular reforms because for a resource limited nation like Nepal, educational interventions are more cost effective [7]. As per the census of Nepal 2021, 24% of the population is adolescents. Moreover, it is the period when habits can be shaped, and students in these grades represent early adolescents, a developmental period where life long dietary behaviors can be established. Therefore, in this study, we have reviewed prevalent nutrition-related disorders among adolescents in Nepal, assessed nutrition education in school curricula (grades 6–8), and proposed evidence-based strategies for implementing nutrition education.

Lastly, this study was carried out to inform policy and programs to develop a curriculum addressing the current gaps in nutrition education in Nepal. 

## Methodology

The PRECEDE part of the PRECEDE –PROCEED planning model, developed by Lawrence W. Green [8] and Taba’s model of curriculum development [9] were applied for identifying gaps in the present curriculum in terms of alignment of objectives, teaching methods and evaluation components. Moreover, PRECEDE model was useful to generate research questions, and then mapping of codes for further analysis of the curriculum (Table 1). Together these two models provided a comprehensive assessment tool of both behavioral and pedagogical dimensions of nutrition education and thus were agreed upon as suitable tools for curriculum analysis by the authors.

**Table 1:** PRECEDE planning model for curricular analysis

Precede Assessments		Research Questions for curriculum evaluation?	Themes/codes
phase1: social assessment		Does the curriculum address social determinants of nutrition?	Triple burden of malnutrition, social determinants, of dietary behaviors among children like poverty and food insecurity.
Phase 2: Epidemiological assessment		Does the curriculum address nutritional problems, among adolescents, consequences of poor nutritional choices.	Anemia, micronutrient deficiencies, childhood and adolescent obesity, risk factors of NCDs.
Phase 3: Educational and ecological assessment	Predisposing factors Enabling factors Reenforcing factors	Does the curriculum address factors influencing dietary behaviours?	Knowledge, attitude, skills, family and peer influences.
		Does the curriculum improve nutrition related knowledge, attitude, beliefs and motivation?	Food groups, healthy eating, nutrition literacy, food taboos
		Does the curriculum provide practical skills, critical thinking skills to adopt healthy behaviors?	Meal planning, reading nutritional labels, selecting foods from food pyramid, creating healthy food plate, cooking meals.
		Does the curriculum mention or involve in any form the family, community systems to reinforce healthy eating and physical activities?	Parent engagement, food environment, school food environment
Phase 4: Administrative and Policy assessment		Does the curriculum include pedagogical, assessment methods and other implementation mechanisms required?	Teacher training, instructional time, assessment strategies, school rules and regulations., policies

The contents were evaluated based on the following research questions based on Taba’s model of curriculum.

1. Are the curriculum’s learning goals challenging, clear, and appropriate; is its content aligned with its learning goals?
2. Is it accurate and appropriate for the intended audience?
3. Is the instructional design engaging and motivating for the intended student population?
4. Is the system of assessment appropriate and designed to guide teachers’ instructional decision-making?

Other sources including Nepal Demographic Health Survey 2016 [10], NDHS 2022 [11], National Nepal Micronutrient Status Survey 2016 [12], and Global Nutrition Report 2022 [13], were reviewed to identify nutrition-related problems in Nepal among adolescents and for verification of information mentioned in the textbooks.

### Curriculum analysis

For the analysis of school curriculum, the Basic Education Curriculum, 2012, and their official textbooks published by Curriculum Development Centre Sanothimi, Ministry of Education, from grade six to grade eight were reviewed [14]. The focus of this evaluation was to analyze the extent and accuracy of nutrition-related content based on the research questions given by the PRECEDE and Taba’s model. Then, curriculum was systematically evaluated to assess the integration of nutrition-related topics in the subjects. The textbooks’ contents were especially mapped by carefully reviewing them to identify in which subjects the themes related to nutrition were included. Besides, the depth and duration of the contents were analyzed as well [15].

Common Consensus on codes and themes was reached among authors through group discussions and relevant literature review. These authors, who are experts from backgrounds- Masters in Public health nutrition (AD), MD physiology (NB), Master’s in Health Education and promotion (BS) and Masters in Education (NB) These authors have 3–10 years of teaching experience in medical colleges. (TMS, BS, AD, NB) were involved in mapping of nutrition related contents in the curriculums.

The content’s accuracy and relevance presented in the curriculum were assessed by factchecking, application to real life, and application beyond the classrooms [16]. Furthermore, the textbook assessments were evaluated for promoting critical thinking capacity and deeper learning opportunities [17]. Based on the above analysis, the strengths and weaknesses of the contents of each grade were listed. The identified gaps were documented. In the next step, we evaluated the nutrition-related curriculum taught in other countries using case study methodology. The evaluation included the curriculums available in English., easily accessible online and, carried the essence of community education. Based on a review of few curricula for other countries, we included two case studies that seemed most relevant to the Nepal situation: Alberta Health Services, Canada, and Community Voices for Health, American University, USA and UK national curriculum to compare with the Nepalese curriculum [18–24] because they had complete structure and were designed by reputable institutions and developed nations. All the curriculum was developed by pioneer countries in education; hence, the curriculums were selected for comparison.

**Fig. 1:**
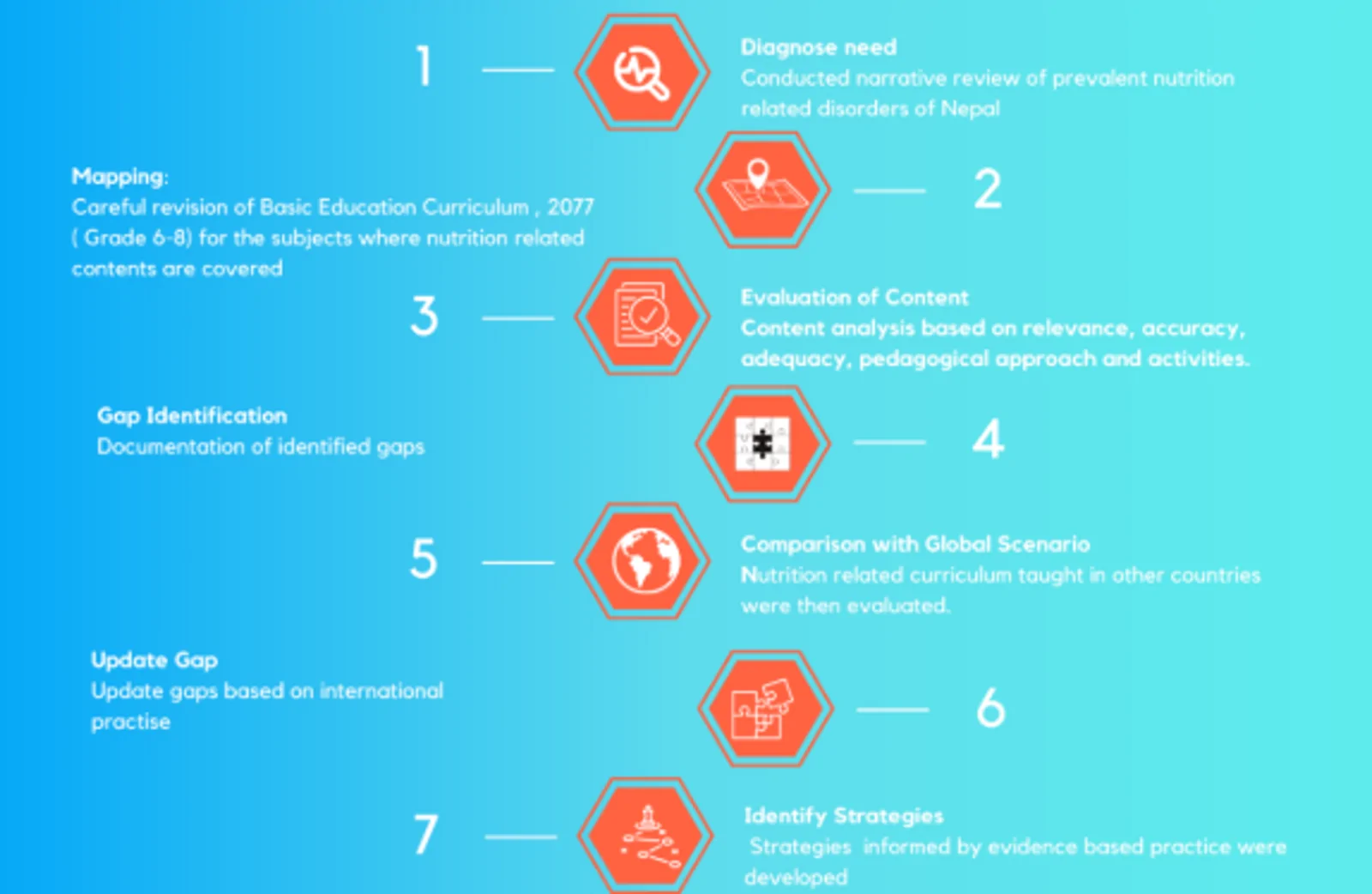
The study design methodology

Comparing our curriculum with international curriculums was pivotal for understanding the level of alignment with global educational practices in terms of nutrition knowledge and skills and teaching learning methods. Based on these differences identified suitable strategies were outlined to address the gaps in nutrition education in school children from grades six to eight in Nepal. The methodology has been summarized in Fig. 1.

## Results

### National nutritional status

Nepal is facing a triple burden of malnutrition. The rise of non-communicable diseases is alarming in the country [25].

Though we have made significant progress in the management of Vitamin A related disorders, stunting and wasting prevalence in the country [26]. However, the consumption of ultra processed foods, calorie dense foods and sweetened beverages, low fruits and vegetables intake is high in the country among school going children. The long-term consequence of these dietary habits is obesity leading to chronic diseases. Knowledge of behavior change can be one of the least expensive interventions to manage the situation but, education to children regarding nutritional choices [27, 28]. Knowledge about foods with vitamins, minerals and proper food choices and meal plans lack tremendously. These micronutrients are even more important among preschoolers and school children for their cognitive development.

Obesity in children accounts for more than 1%. Folate deficiency is 6.2% in children. 17.2% of children have Vitamin D deficiency and 43. 1% of children have vitamin B6 deficiency. Nearly 60% of children are selenium deficient. These deficiencies could translate to consequences later in adulthood in the forms of neurodegenerative diseases and risk of cardiovascular diseases. Moreover, an estimated 2–3% of GDP is spent due to vitamin and mineral deficiencies in Nepal. These deficiencies are associated with long term chronic diseases’ risks and developmental consequences and cognitive problems [29, 30]. Nepal Demographic Health Survey Report 2022 reports that stunting and wasting(25%, 8%) and have slightly reduced from the situation of 2016 (36%, 10%respectively) [11] but, we are still behind commitments of Sustainable Development Goals [31].

### Nutrition education in Nepalese school curriculums

In grades six, seven and eight, the food and nutrition contents in Science and Health and Population subject [14, 15] were analyzed and the findings have been presented in Tables 2, 3 and 4 in detail respectively. We noted that the curriculum introduces fragmented nutrition concepts to these students, lacks practical relevance and pedagogical underpinnings. The activities designed had minimal reflective learning and critical learning opportunities. The lessons were limited to chapters and disconnected from daily life.

The strengths of Nepal’s curriculum are: foundational concepts to nutrition (food groups, nutrients), progressive complexity across grades, few culturally relevant foods mentioned and hands-on activities. Tools that are not reflective of current public health priorities such as the food pyramids rather than food plate were introduced in those textbooks. There were limited opportunities to develop life skills such as portion size, identifying junk foods, recognizing misleading advertisements, over nutrition, basics of cooking etc. Although students were introduced to limited concepts about junk food, it was far from developing real nutritional awareness.

There are plenty of examples of misinformation regarding food like presence of greens in flavored instant noodles and malted drink powders boosting growth in height [32]. Sugar based cereals are marketed as healthy breakfast. Children easily believe these false claims. Therefore, as educators we believe that the outcome of nutrition education at school level is to empower students to make informed, safe, and independent food choices. Regrettably, the school curriculum is not designed adequately communicate nutrition education to cater their needs.

**Table 2:** Evaluation of Nutrition Education for Grade 6

Strength	Weakness
• Dedicated Nutrition Chapter: A dedicated nutrition chapter is included in the curriculum, emphasizing the importance of nutrition in education. Macronutrients (carbohydrates, proteins, fats and their sources are well introduced) • Clear Categorization of Foods: Students can understand the contents as simple categorizations of food have been made. • Cultural Relevance: Culturally relevant foods and Nepali names enhance the relevance of the curriculum within the local context. • Basic Framework Introduction: A basic framework for dietary requirements, energy-giving, bodybuilding, and body-protecting foods will provide the foundation for nutrition knowledge. • Fundamental Exercise: The exercises in the textbook provide students with a fundamental understanding of nutrition concepts. • Physical exercise is incorporated with nutrition-related information. Steps for doing exercise are also taught. • A separate chapter on Yoga and physical exercise is kept. With pictures of yoga and exercise poses.	• Information regarding micronutrients, vitamins and minerals is lacking. • Lack of Depth: Although the content introduces nutrition, a deeper understanding fails to develop, potentially leaving students unprepared. • Missing Portion Guidelines: For understanding a balanced diet, there is a lack of information on portions and nutrients such as fat. • No Balanced Food Plate: Lack of a simple and easy “food plate” model • Absence of Real-Life Examples: A lack of real-life examples and practical applications makes it difficult for students to apply their knowledge in everyday situations. • Limited Visual Aids: Visual aids lack naming and descriptions, which could make it difficult for students to use them effectively. • Overemphasis on Recall: Textbook exercises tend to focus on simple recall rather than fostering critical thinking and deeper understanding. • Assessment Gaps: Students aren’t assessed on how their nutrition knowledge can be applied in everyday life, which creates a gap for real-life application. • Lack of Reflective Exercises: Less emphasis is placed on students reflecting on what they have learned, which inhibits critical thinking. • Limited Question Diversity: Question types are limited to comprehensively assess students’ skills and knowledge. • Missing Practical Skills Lessons: The curriculum does not address practical skills such as gardening, breastfeeding, cooking, and address obesity and undernutrition, which are vital to real-life situations. • Content accuracy is lacking in many places (For e.g.: Jaundice is introduced as the presence of virus in blood)

**Table 3:** Evaluation of Nutrition Education for Grade 7

Strengths	Weakness
• Graded Content: Grade 7 content is more challenging than Grade 6 content, which encourages students to learn more. • Deeper Understanding: The curriculum introduces the food pyramid and categorizes foods according to dietary needs. • Visual Learning: Students are more likely to comprehend complex concepts when they use diagrams and images. • Nutrient Detail: Nutrients’ sources and functions are discussed in detail. • Awareness of Junk Foods: Learning about junk foods allows students to realize the importance of a healthy diet. • Hands-On Activities: Practical activities are included, e.g. collecting information about junk foods available in the market, bringing foods from home, and discussing their nutritional value.	• Superficial Learning: Despite its graded difficulty level, this content promotes superficial learning, particularly about nutrients and sources. • Complex Food Pyramid: Complex food pyramids may overwhelm students. Further, the food temple released by the Dietary Guidelines of Nepal is not incorporated. • Lack of Standardization: There are no specific guidelines for evaluating nutritional values in activities, which leaves room for misinterpretations. • Rote Learning: Practices such as matching exercises and short answers may encourage rote learning, reducing opportunities for deeper understanding. • Exclusion of culturally relevant Important Topics: such as ghee are not covered in the curriculum, which limits students’ knowledge.

**Table 4:** Evaluation of nutrition education of Grade 8

Strengths	Weakness
• Dedicated Nutrition Chapter: There is a separate chapter dedicated to nutrition education but was found only in health education subject. • Coverage of Nutrition Disorders: • Nutrition-related disorders like rickets, anemia, night blindness, and scurvy are well covered. • Symptoms and Preventive Measures: The signs and symptoms of these disorders offer practical preventive measures, enhancing students’ understanding. • Balanced Diet: A balanced diet, and locally produced food are encouraged. • Practical skills like the preservation of nutrients are given.	• Confusing Texts: and confusing irrelevant sentences, such as enhancing awareness of marasmus in the context of night blindness. • Subjects like science and social studies had no mention regarding food and nutrition. • Content accuracy was not met which can lead to frequent misunderstanding among children. (Example: Malnutrition means lack of carbohydrates; anemia is caused because of iron and vitamin C deficiency) • Lack of Clarity: It should follow a logical and comprehensible structure for students in presenting complex ideas on nutrition-related disorders. • Superficial Approach: While discussing complex topics like malnutrition, the curriculum has a superficial approach, which may hinder students’ understanding. • Examples like wrong eating habits lead to malnutrition but wrong were not described with examples. • Inadequate Visuals: Limited images and visuals to aid learning as visuals are essential for comprehensive learning at the school level. • Missing Cognitive and Emotional Aspects: Integration of the cognitive and emotional components, is lacking. These aspects are important for wholesome learning. • ability to apply their knowledge and skills. • Inadequate information regarding Processed Foods: The curriculum doesn’t sufficiently address processed foods, including their definition, examples, and their impact on health.

These comparisons are presented in Table 5. On review we observed that the curriculum adopted in Nepal is traditional, lacks depth and standardization. In line with our observation, the life skills content was limited. The other two curricula outcomes were also focused on developing healthy behavior and life skills. The terms used may not be readily understood by students, such as cereals which could be replaced by grains. Portion sizes, practical applications, and socially relevant examples are lacking. As we do not consume food as isolated categories but as a whole dish, illustrating foods in their real form becomes imperative, such as rice pudding, dumplings, thali etc. This assures that nutrition education becomes more relatable and easier to understand. In contrast, the curriculum developed by Alberta Health Services [22] was structured and student friendly. There were clear nutrition groups such as grains, fruits, milk, vegetables, and meat. The curriculum also suggests alternatives. The content which was not available in Nepal’s curriculum was: portion sizes, serving examples, whole grains and fiber rich foods and overconsumptions. The activities designed were to think critically and practically along with reflective activities.

**Table 5:** Comparison of Nepalese Curriculum with International curriculums

Contents	Examples (where relevant)	Nepal	Alberta Health Services, Canada	Community Voices for Health, American University, USA	UK government (England and Wales)
Categories of food		Cereals, beans & pulses, fruits & vegetables, water, milk, fish & meat	Grains, vegetables & fruits, milk and alternatives, meat & alternatives	Carbohydrates, proteins, fat & oil, minerals, Vitamins, and Water	Carbohydrates, fats, proteins, fibers, vitamins, minerals.
		Remark: Cereal is often a confusing term for children as many believe it to be corn flakes and chocolate flavored sweetened breakfast foods.	Remark: Grains are clearer for students to grasp.	Since the curriculum is integrated, the categories are based on science.	Integrated curriculum. Taught across vaious subjects like biology, Design and Technology, science.
Concept of a balanced diet		Only type	Appropriate amount (quantity) and type (quality) of food.	Appropriate amount (quantity) and type (quality) of food.	Amount and portion size of food mentioned. And is aligned with government recommendation(Eat well guide.)
		Remark: Lists of foods provided	Remarks: The curriculum includes detailed information about portion sizes and specific examples of foods within each category. Here’s an example of how such information might be structured for the “Grains” category: 1 slice of whole wheat bread or ½ cup of cooked white pasta	Remark: Typical serving sizes provided.	
Knowledge about diversified foods		✩✩✩	✩✩✩✩	✩✩✩	✩✩✩✩
		Remark: Names listed out	Remark: Plenty of lists along with serving sizes provided	Remark: Typical foods and their serving sizes are provided.	Typical foods and their serving sizes are provided. Also includes meal planning classes.
Knowledge about food safety and hygeine		Present	Present	Present	Present
Over consumption nutrients		Acknowledges obesity as a problem	Acknowledges effects of overconsumption of nutrients.	Acknowledges effects of overconsumption of nutrients.	Acknowledges long term effects of childhood obesity.
Alternatives	Evaluating different meal options, comparing snack choices	Doesn’t include	Included	Doesn’t include	Included
Healthier food options such as whole grains and fibers		Doesn’t include the incorporation of whole grains into diet	Clearly explains about whole grains and fibers	Clearly explains about whole grains and fibers	Clearly explains about the importance of fiber, water and minerals and trace minerals.
Healthy behavior	(E.g: physical activity for obesity management, avoidance of sweetened beverages)	Doesn’t include.	Included	Doesn’t include	Included
Adolescent Nutrition		Not mentioned	Included	Included	Nutrition across life span including adolescence.
Standardization:	systematic progression and uniform in detail guidelines, validation of content and accuracy	Lacking	Present	Present	Present
Relevant activities to real-life like	(school kitchen -gardening, art and craft)	Lacking	Present	Present	Present( schools teach students knife skills and cooking classes are given, meal planning is taught)
Reflective activities like	reflective writing, maintaining a food diary	Present but minimal.	Present	Present	Present
Deep learning and critical thinking activities like dedicated course on nutrition in secondary level.	Analysis of food labels, identification of misinformations,	Lacking	Present	Present	Present
Visual representation of a balanced diet		Food pyramid.	Food plate.	Food plate	Food plate
		Remark: Recall-based activities	Remark: Critical thinking activities	Remark: Critical thinking activities	Remark: Critical thinking activities
Learning opportunities related to life skills	Meal planning, cooking skills, reading food labels	Lacking	Present	Lacking	Present
			Remark: self-perception, self-esteem, and role of healthy behaviors.		Remark: food choices, meal planning.
Nutrition label		Lacking	Lacking	Present	Present
Content Integration		Present in grades 6-7-8 in health education subject	Present	Present	Present( in key stages across all subjects)
Content Accuracy		✩✩✩	✩✩✩✩✩	✩✩✩✩✩	✩✩✩✩✩

The Canadian curriculum developed by Community Voices for Health curriculum [23] uses an integrated curriculum model. Foods were categorized through scientific concepts such as carbohydrates, proteins, fats, vitamins, and minerals. This helps link nutrition with biological understanding. Like curriculum by Alberta, the curriculum introduces serving sizes, overconsumption and food plate. The curriculum was standardized, filled with reflective activities, and opportunities for deep learning. A unique feature of this curriculum was the inclusion of affect components such as self-perception, self-esteem and healthy behaviors modeling. Unlike the other two, it includes nutrition labels and teaches students how to interpret them which is an essential real-life skill. The key findings are that there are clear differences in international curriculums and the school level curriculum of Nepal. The contents of these two international curriculums have quantified the amounts of diets required for the children including the portion sizes but that of Nepal has only defined what a balanced diet is. The international curriculums have mentioned the effects of overnutrition, but the Nepali curriculum has not mentioned it at all. The other significant difference that we found is that that the curriculums of Canada and USA have mentioned about dietary fibers and whole grains but that is not found in Nepali textbooks. Moreover, these curriculums emphasize reflective learning, systematic progression of knowledge and fostering real life skills which are necessary to embrace healthy behaviors early in life.

Based on our review and analysis of the Nepal nutrition education curriculum and its comparison to international curricula, strategies for improving nutrition education in Nepal have been identified and listed as:

1. Integrated curriculum: Incorporate nutrition education into other subjects, such as science, health, physical education, and math (e.g., calculation of nutritional values) as part of an integrated curriculum. This approach emphasizes nutrition in all aspects of one’s life.
2. Project-based learning: Encouraging healthy behavior and developing a deeper understanding of nutrition concepts through project-based learning (PBL) in schools [15]. For e.g.: Farm-to-school.
3. Affective and Behavioral aspect: Design learning objectives incorporating affective components (motivations, values, etc.), and personalized behavioral components ( self-assessments, behavioral-change strategies like cook your meals session in schools, food tasting in classrooms [19].
4. Providing adequate learning opportunities: One unit lesson each year limits students’ abilities to develop informed nutritional decisions. To achieve better learning results, a competency-based approach is recommended without time constraints. The focus of content should be what students learn deeply and apply practically at the end of each chapter and not what students are to be taught. The assessment should be based on real demonstrations. Eg. Evaluate your lunch/dinner and identify whether it is balanced [33].
5. Parent and community involvement: Parental modeling and an enabling environment in the community are essential to the development of nutrition behaviors. For e.g. Build your school gardens, sharing ethnic recipes, local farmer’s market visits with parents etc. [20]
6. Coordinating with school meal programs: Cafeterias can serve as learning labs, in which students are exposed to balanced meals [17].
7. Reflective activities: Students are encouraged to become aware of their food choices through this activity. Eg. food diary, food photographs [18],
8. Activities that are more relevant and effective based on the real world: For e.g. My plate, reading nutrition labels, meal planning, portion analysis, and planning, recognizing junk foods.
9. Toolkit for teachers to teach nutrition education.
10. Giving priority to culturally and locally relevant foods and their health benefits.
11. Include content related to body image, self-esteem building, and the adverse effects of poor behavior related with food choices.
12. Present food is more than just a source of fuel and nutrients. Teach how food provides pleasure and connects us with ourselves, our families, our cultures, our nature, and our communities [33].
13. Ensure that learning resources for students are accurate by involving multiple stakeholders, such as nutritionists and health professionals.
14. Introduce problems associated with overnutrition in subjects like science or introduce a health and nutrition as a separate subject or a module.

## Discussion

Our analysis reveals significant shortcomings in Nepal’s school curriculum when compared both national needs and global nutrition education practices. Although nutrition topic was introduced to each grade, the content and pedagogical approach is inadequate to influence dietary behavior change.

The national survey suggests high levels of anemia, micronutrient deficiencies, childhood obesity in Nepal [29]. A study done in Pokhara in 2024, Nepal showed that 70% of students consumed junk foods three or more times weekly [34]. However, we observed limited priorities given to these topics. We strongly think the adult life consequences of these conditions for example linking obesity with cardiovascular diseases, diabetes, should be briefly introduced in this level because only the “ what part” does not enforce behavior change. The content was focused on superficial text rather than conceptual relevant learning. The contemporary nutritional challenges such as overconsumption of sugar and salt, health misinformation, misleading marketing, obesity are not touched upon in textbooks. These are huge influences for children to eat sweets, cold drinks and from unhygienic street foods contributing to their poor health [35, 36].

### Pedagogical limitations

Pedagogical theory suggests that to bring a behavior change the education imparted should support reflection, critical thinking and skills. But, The key limitation in our textbooks was the absence of a clear vision for fostering behaviour change in the students while designing the curriculum. We acknowledge that the purpose of nutrition education is not merely memorizing facts, but to shape student’s behaviour and enable them to make informed decisions. We observed the curriculum siloed with heavy text, rudimentary and disconnected from Nepalese societies. The Nepal curriculum focused on rote memorization, limited opportunity to reflect, under addressed affect components of learning. The curriculum provides information but lacks behavioral and affective scaffolding. To address this, we can take notes from some randomized controlled trials that have shown successful results in increasing students’ preferences for fruits and vegetables through food tasting, building school kitchen gardens and cooking classes as interventions and integrate these in our curriculums as well [36–38].

Further, the use of outdated instructional models such as the food pyramid and few visuals, limits opportunities to connect nutrition education to daily lives. The curriculum is required to be relatable to students; hence, sparsely discussed ethnic foods, foods presented in categories rather than in dishes, etc., make it difficult for students to comprehend [38, 39] Even more unfortunate is that the curriculum development center of the country has not made health and population a mandatory course for students in grades 9 and 10 in Nepal. This limits the learning opportunity of students at the secondary level regarding nutrition and health.

### Comparison with global practices

We found that the curriculum developed by Alberta Health Services, Canada [22], and Community Voices for Health, American University, USA [23] were standardized, detailed and followed conceptual logic. Nutrition was presented in progressive manner in increasing depth. The pedagogical exercises included reflective activities at the end of each session, assisting learners to select appropriate portion sizes, make their own balanced food plates, and devise alternatives. Also, the curriculum focused on the affect components of nutrition education, such as self-esteem, body image, etc. The policy makers must be informed regarding such concerns. While revising existing curriculum, they could integrate topics such as emotional eating, self-esteem, encountering social pressure, cultural aspects, etc. to create enabling and reenforcing environments for children to choose healthy choices leading to healthy lifestyle.

**Fig. 2:**
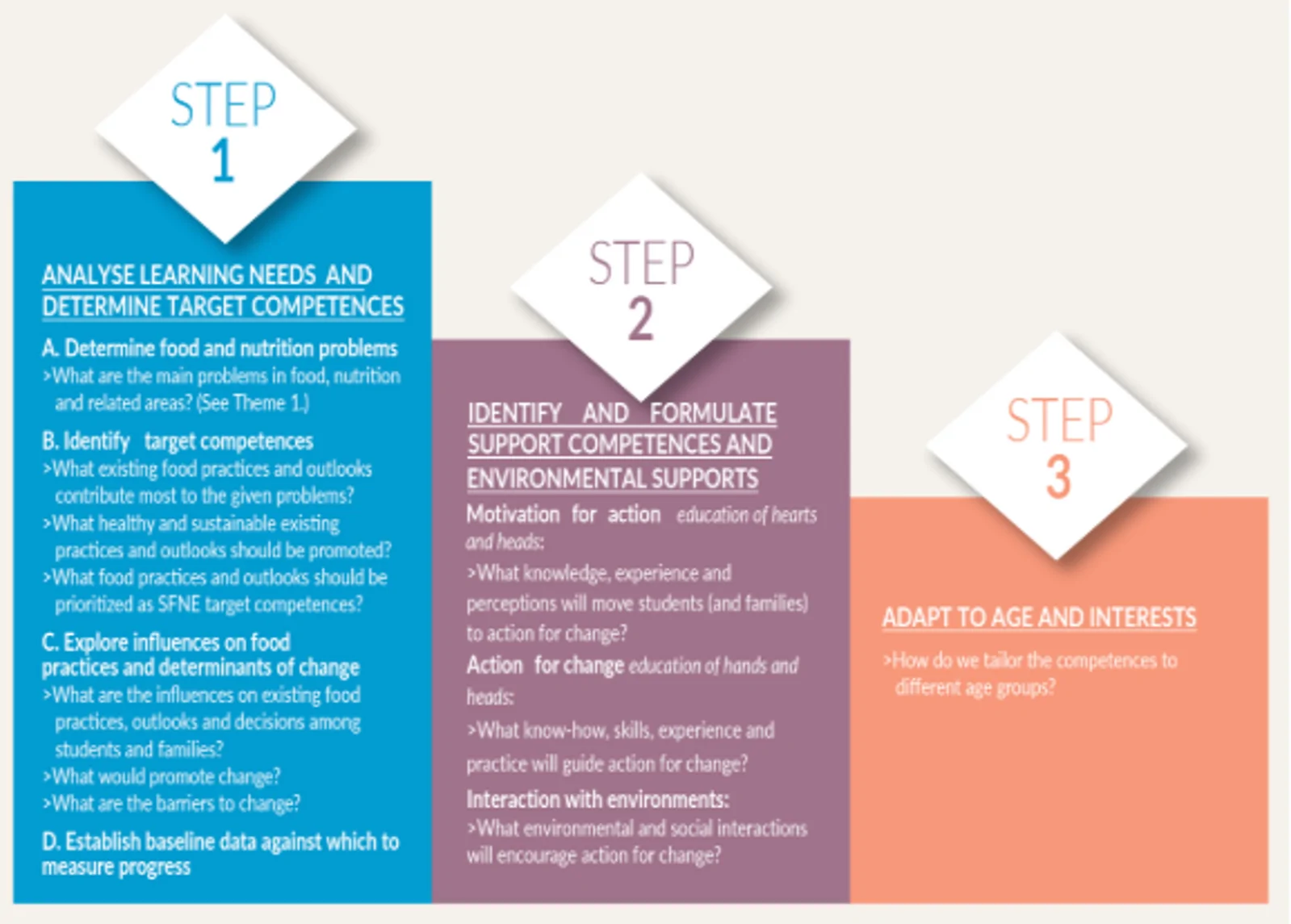
The framework for identifying and formulating nutrition education

For imparting nutrition education to bring sustainable behavior changes, we suggest a framework proposed by Contento et al. [36]. This has also been suggested by FAO and has been elaborated in Fig. 2. We believe this is appropriate for Nepal because it recognizes behaviors related to nutrition are determined by both external factors (social structure) and internal factors, i.e., psychological process ( motivation).

According to the proposed framework, problems should be identified by analyzing the current situation. After evaluating existing practices and perspectives, reviewing educational priorities within the country, and developing key competencies for children, the process involves establishing key competencies for the children. Moreover, it requires an understanding of the learning that enhances motivation or facilitates change, and the environmental conditions that allow for an effective implementation.

**Fig. 3:**
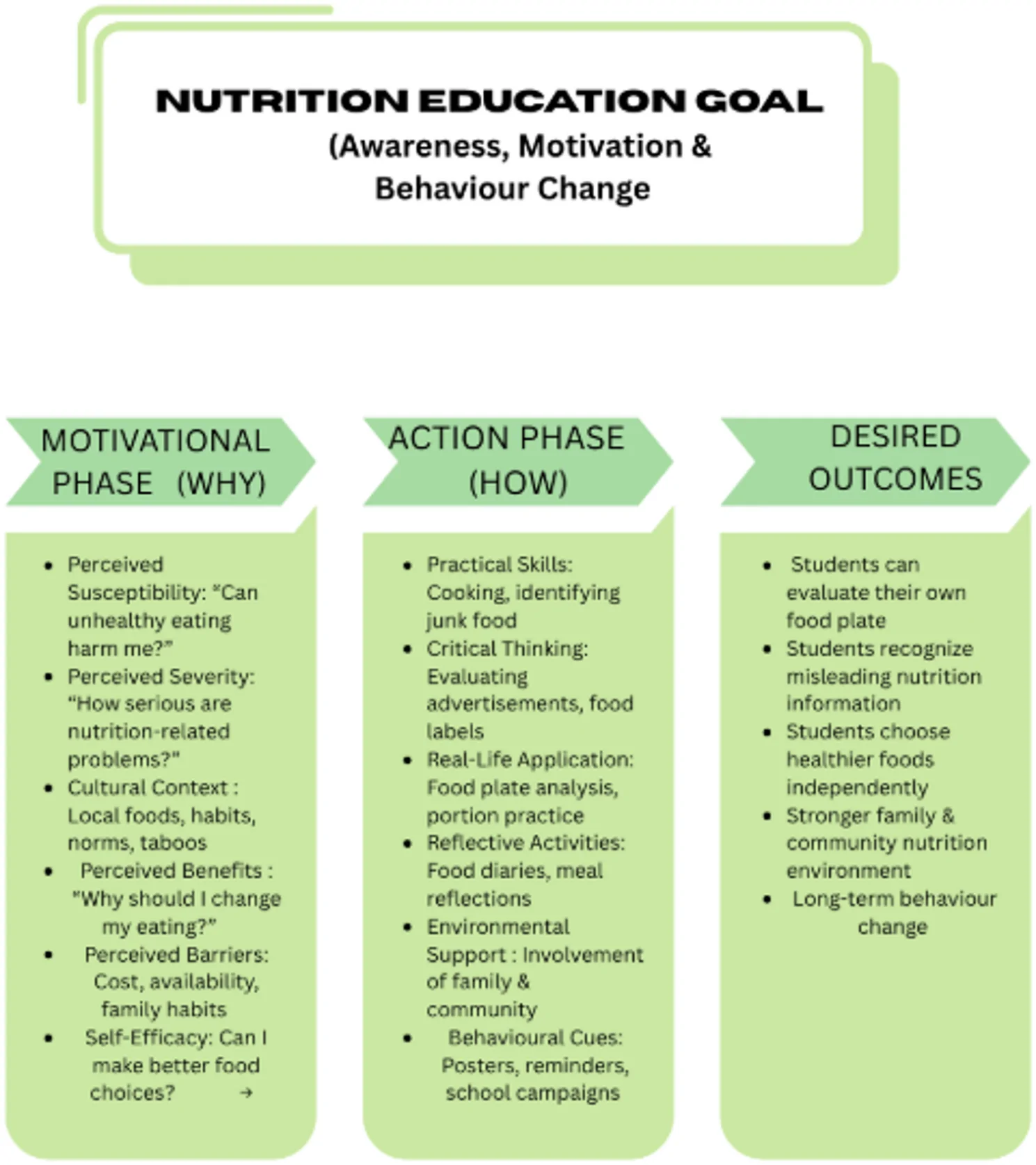
Conceptual framework for nutrition education using Health Belief Model

The authors have presented a preliminary conceptual framework (Fig. 3) for further developing and changing the Nepal nutrition education curriculum using the Health Belief Model [40]. The authors recommend focus group discussions and interviews with public health educators to further refine and redefine nutrition education in Nepal for school children. In this figure developed by Jones et al. ( Fig. 3), we see connections between the “why” and the “how”. The “why” pertains to the reasons behind individuals’ beliefs and perceptions about nutrition, representing the motivational phase of the behavior change process [36]. Cultural context has a significant impact on the motivational phase since it influences perceptions and beliefs. Hence, when designing the motivational phase for students, cultural considerations need to be taken into account. Similarly, “how” refers to the action phase of behavior change, which includes strategies and interventions intended to foster behavioral change in students. In this regard, the goal of nutrition education can be defined as enhancing awareness and motivation and facilitating individuals in taking proactive actions regarding nutrition.

### Implications for research and policy development

It is crucial to update the curriculum to empower students to make informed nutritional choices. The following are some next steps to improve student learning about nutrition:

- Increase stakeholder engagement, including students and parents, in curriculum development.
- Explore evidence-based teaching strategies that foster critical thinking and real-life application of nutritional concepts.
- Incorporate local and contextual culinary knowledge into the curriculum and explore its effectiveness.
- Advertise Junk food as unhealthy for growth and development of physical and mental health.
- Adoption of project-based learning approaches,
- Incorporating behavioral change aspects, contextualized learning, replacing nutrition pyramid with my plate, reflective activities such as food diary, affect component, engaging parents and communities in the process.
- Promote audio visual aids for instructional purposes and collaborating with nutritionists to develop educational resources.
- Research for developing educational techniques and empowering teachers is also a must.

## Limitations

Due to certain geographical and financial restrictions, we could only limit our study to a content analysis which is a qualitative review and could not spend time with students, teachers and education policy makers for an open policy level research or discussions to understand the processes how nutrition knowledge is being integrated in real classrooms and what is actually feasible for them. Also, more references from other countries should be included in this process of analysis to draw more pedagogical interventions for effective behaviors change which lacks in our paper. We acknowledge that two of our curricula’s references(Alberta, Canada and America) are not official government curriculums and only one of them (UK ) is official and this might have not justified their comparison with national curriculum of Nepal. But we believe these unofficial curriculums were able to capture sense and needs of the community-based curriculums. We highly encourage researchers and health educators to bring more evidence on creative pathways for integration of nutrition knowledge in curriculum and classroom activities in the future. Such research could provide more specific solutions to educate our children about nutrition and health.

## Conclusion

An enhanced curriculum needs to be relevant as well as accurate while also offering opportunities for in-depth learning. This content analysis suggests strategies for an upgraded curriculum, such as integrating nutrition education across multiple subjects.

This revised curriculum should prioritize behavior change towards healthier nutrition practices, aiming for a significant decrease in nutrition-related disorders prevalent or could increase in the days to come.

## Data Availability

The datasets used and/or analysed during the current study are available from the corresponding author on reasonable request.
